# Genetic dissection of grain traits and their corresponding heterosis in an elite hybrid

**DOI:** 10.3389/fpls.2022.977349

**Published:** 2022-10-05

**Authors:** Sundus Zafar, Hui You, Fan Zhang, Shuang Bin Zhu, Kai Chen, Congcong Shen, Hezhou Wu, Fangjin Zhu, Conghe Zhang, Jianlong Xu

**Affiliations:** ^1^ Shenzhen Branch, Guangdong Laboratory for Lingnan Modern Agriculture, Agricultural Genomics Institute at Shenzhen, Chinese Academy of Agricultural Sciences, Shenzhen, China; ^2^ The National Key Facility for Crop Gene Resources and Genetic Improvement, Institute of Crop Sciences, Chinese Academy of Agricultural Sciences, Beijing, China; ^3^ Hunan Tao-Hua-Yuan Agricultural Technologies Co., LTD., Hunan, China; ^4^ Win-All Hi-Tech Seed Co., Ltd., Hefei, China; ^5^ Hainan Yazhou Bay Seed Lab/National Nanfan Research Institute (Sanya), Chinese Academy of Agricultural Sciences, Sanya, China

**Keywords:** grain shape, grain weight, grain yield, quantitative trait locus/loci (QTL), heterosis, pyramiding breeding

## Abstract

Rice productivity has considerably improved due to the effective employment of heterosis, but the genetic basis of heterosis for grain shape and weight remains uncertain. For studying the genetic dissection of heterosis for grain shape/weight and their relationship with grain yield in rice, quantitative trait locus (QTL) mapping was performed on 1,061 recombinant inbred lines (RILs), which was developed by crossing *xian*/*indica* rice Quan9311B (Q9311B) and Wu-shan-si-miao (WSSM). Whereas, BC_1_F_1_ (a backcross F_1_) was developed by crossing RILs with Quan9311A (Q9311A) combined with phenotyping in Hefei (HF) and Nanning (NN) environments. Overall, 114 (main-effect, mQTL) and 359 (epistatic QTL, eQTL) were identified in all populations (RIL, BC_1_F_1_, and mid-parent heterosis, H_MP_s) for 1000-grain weight (TGW), grain yield per plant (GYP) and grain shape traits including grain length (GL), grain width (GW), and grain length to width ratio (GLWR). Differential QTL detection revealed that all additive loci in RILs population do not show heterotic effects, and few of them affect the performance of BC_1_F_1_. However, 25 mQTL not only contributed to BC_1_F_1_’s performance but also contributed to heterosis. A total of seven QTL regions was identified, which simultaneously affected multiple grain traits (grain yield, weight, shape) in the same environment, including five regions with opposite directions and two regions with same directions of favorable allele effects, indicating that partial genetic overlaps are existed between different grain traits. This study suggested different approaches for obtaining good grain quality with high yield by pyramiding or introgressing favorable alleles (FA) with the same direction of gene effect at the QTL regions affecting grain shape/weight and grain yield distributing on different chromosomes, or introgressing or pyramiding FA in the parents instead of fixing additive effects in hybrid as well as pyramiding the polymorphic overdominant/dominant loci between the parents and eliminating underdominant loci from the parents. These outcomes offer valuable information and strategy to develop hybrid rice with suitable grain type and weight.

## Introduction

Rice (*Oryza sativa*) is an important staple food globally, feeding about half of the world’s population. To fulfill the increasing feeding demand of the people, more attention should be paid to developing high-yield rice varieties (Ikeda et al., 2013). However, with the development of the economy and living standards, grain quality has also becomes the prime objective of breeding, along with high yield (Zou et al., 2018; de Oliveira et al., 2020). Grain weight is commonly evaluated by 1000-grain weight (TGW) that contributes to grain shape and yield traits, respectively (Qiu et al., 2017; Xia et al., 2017). Rice yield and grain quality are also determined by grain shape and weight (Yu et al., 2018). Thus, they significantly play an important role in rice breeding program because of their contribution to rice yield and quality.

The complex genetic mechanism of multiple grain traits is regulated by QTL, which locates on all 12 chromosomes (http://www.gramene.org/). Over the last decades, QTL mapping has been widely used to analyze grain shape quality traits (Bai et al., 2010; Han and Huang, 2013; Huang et al., 2013). By using different mapping populations (F_2_, BC_1_F_1_, RIL, double haploid, DH), many QTL related to grain traits were identified (Huang et al., 2013; Kabange et al., 2020; Li et al., 2021). A few genes were cloned among them like *GW2* (Song et al., 2007), *GW5* (Liu et al., 2017), *GW6* (Shi et al., 2020), *GW8* (Wang et al., 2012), *GS3* (Mao et al., 2010), GS5 *(* Li et al., 2011), GS2 *(* Che et al., 2015 *;* Hu et al., 2015), *GS9* (Zhao et al., 2018), *GLW7* (Si et al., 2016 *)*, *GL4* (Wu et al., 2017), *GL6* (Wang et al., 2019a), and *LGY3* (Liu et al., 2018), which regulates grain weight and shape. Combining favorable alleles (FA) of these genes can enhance rice quality and yield. For instance, introgression of different alleles of *LGY3* and *GS3* into the indica variety RD23 can attain different yield-increasing effects, and pyramiding of *LGY3* and *GS3* in RD23 proved to be 10.9% more productive with improved grain quality than pyramiding of *LGY3* and *GS3* (Liu et al., 2018). Currently, single nucleotide polymorphisms (SNPs) are preferably used because of their abundant presence (Kumar et al., 2012), while next-generation sequencing (NGS) permits the detection of several SNPs for different plant species. Based on SNP marker, a high-density map can be prepared, which can improve the accuracy/efficacy of QTL mapping (Li et al., 2014).

Heterosis, is a phenomenon, in which F_1_-hybrids show far superior phenotypic performance compared to their parents (Chen et al., 2018). F_1_-hybrid plants have noticeably increased grain yield, biomass production, vegetative growth rate, and stress tolerance (Fujimoto et al., 2018). Many hypotheses related to heterosis have been proposed as heterosis plays a crucial part in crop genetic research, such as dominance, over-dominance, and epistatic hypothesis (Hochholdinger and Hoecker, 2007; Goff and Zhang, 2013). Despite being broadly exploited in plant breeding, the molecular mechanism of heterosis is still poorly understood (Bagheri and Jelodar, 2010 **;** He et al., 2010; Kim et al., 2017). Generally, heterosis is regulated by several genes, epistasis, and gene-by-environment interaction (Li et al., 2015; Li et al., 2018; Lin et al., 2020a). Researchers have extensively studied the heterosis in rice (Zhou et al., 2012 **;** Xiang et al., 2016), but less attention has been paid to multiple rice grain traits.

A high-yielding superior Quan-you-si-miao (QYSM) hybrid derived from Q9311A and WSSM has good quality and yield, therefore it is essential to elucidate the genetic mechanisms/relationship of heterosis for multiple rice grain traits. Herein, our objective was to dissect the genetic basis of heterosis for grain weight/shape, and their heterotic relationships with grain yield using the same sets of the RIL and BC_1_F_1_ populations. We detected 126 QTL for multiple grain traits (grain type, weight, and yield) along with previously reported genes in these QTL. Besides, the heterotic effect of QTL for multiple grain traits (shape, weight, and yield) in RILs, BC_1_F_1_s, and H_MP_s was investigated to study the relative significance of both additive/non-additive effects on heterosis. Thus, our findings provide guidance about the genetic basis of heterosis for multiple grain traits that offers supportive information for effectively manipulating the identified QTL to improve the rice grain quality and yield.

## Materials and methods

### Experimental materials

QTL mapping was performed using 1,061 F_8_ RILs derived from the cross of Quan9311B (Q9311B) and Wu-shan-si-miao (WSSM) by single-seed descent, and the corresponding 1061 BC_1_F_1_ progeny developed by crossing the RILs with Quan9311A (Q9311A) (**Supplementary Figure 1**). Q9311A is a three-line sterile line, near-isogenic to Q9311B. Thus, Q9311B was used to cross with WSSM for maintaining normal fertility in the RILs and Q9311A was used to cross with the RILs for easy development of a testcross population. Other materials included an F_1_ hybrid, Quan-you-si-miao (QYSM), which was developed from Q9311A and WSSM, two parents Q9311B and WSSM, which are regarded as backbone parents in hybrid rice breeding programs in China for their superior general combining ability and grain quality.

### Field experiment and trait evaluation

The RIL and BC_1_F_1_ populations were produced in the experimental field of Chinese Academy of Agricultural Sciences, Sanya (18.3°N, 109.3°E), Hainan province, in the 2018 winter season. The RILs, BC_1_F_1_s, parents (Q9311B and WSSM), and QYSM were sown in the seedling nursery on May 17, and July 12, 2019 in Hefei (32.3°N, 117.6°E) of Anhui province and Nanning (22.1°N, 107.5°E) of Guangxi province, respectively. Owing to its infertility, Q9311A was not included in the field experiment for traits evaluation, and its values were evaluated using the near-isogenic line Q9311B. The 25-day-old seedlings were transplanted into a four-row plot with six plants in each row at 13 × 30 cm spacing for two replications in a randomized complete block design. Field, water, weed, and fertilizer management followed to local practice.

At the mature stage, seeds from six uniform plants in the middle of each plot were harvested at the mature stage and air-dried for three months in drying houses to a 13.5% grain moisture content. Post eradicating the unfilled grains, the grains were weighted to calculate grain yield per plant (GYP, g). Grain shape and grain weight traits: grain length (GL, mm), grain width (GW, mm), grain length-width ratio (GLWR), and 1000-grain weight (TGW), were measured with a scanning machine (SC-E, Wanshen Technology Company, Hangzhou, China). All measurements were performed with two replicate samples, and the mean was used for data analyses.

### SNP genotyping

Genomic DNA for SNP genotyping was isolated from approximately 100-mg fresh leaf samples of 5-week-old seedlings for each of the 1061 RILs, Q9311B and WSSM using a modified cetyltrimethylammonium bromide method (Murray and Thompson, 1980). Whole-genome shotgun sequencing of the parental lines (Q9311B and WSSM) and RILs generated 150 bp paired-end reads consisting of ~2.4 Tb of sequence. The RILs were sequenced with ~10x genome coverage, and the two parental lines were sequenced with ~40x genome coverage using the Illumina sequencing platform Novaseq 6000 (CapitalBio Technology Inc., Beijing, China). The sequence reads of the parental lines, and RILs were aligned against the Nipponbare reference genome (IRGSP 1.0) (Kawahara et al., 2013) with BWA package 0.7.1 using default parameters, and PCR duplicates were removed by the ‘MarkDuplicates’ module in the Picard tools (version 1.119) (Li and Durbin, 2009). The raw reads were also re-aligned to identify highly polymorphic regions using the ‘IndelRealigner’ function in GenomeAnalysisTK 3.4.0 (DePristo et al., 2011). Sequence variants between the parental lines were called with ‘UnifiedGenotyper’ in GenomeAnalysisTK. Only uniquely mapped reads were used for subsequent SNP calling. Genotype calling of each RIL was performed based on the SNP alleles between the parents. A total of 741,928 homozygous SNPs polymorphic between Q9311B and WSSM were used for SNP calling in the 1061 RILs. The SNPs were further filtered by removing minor allele frequencies < 0.01 or missing rates > 0.2, leaving 156,373 high-quality and evenly distributed SNPs, located within the genic regions of 15,043 genes based on the gene annotation of the Nipponbare RefSeq from RAP-DB (released on June 26, 2019) (Sakai et al., 2013). A set of 1196 genes was removed because one of the two parental alleles was rare or showed a high frequency of the heterozygote in the RIL population. Then, an allelic genotyping dataset containing two genotypes (Q9311B type and WSSM type) at each of 13,847 genes was generated to be used for bin map construction for the 1061 RILs.

### Bin map construction and QTL mapping

A bin map is a type of genetic map constructed using bin (binning of redundant markers) genotypes, which can save running time of linkage and QTL mapping by greatly reducing numbers of redundant markers. The bin map of the RIL population was constructed based on the two parental genotypes at 13,847 genes using the BIN function in IciMapping QTL version 4.2 (Meng et al., 2015). To remove redundancy, only a single gene was retained to represent each bin, either one gene with a minimum missing rate or random gene when the missing rates were equal. Genes with unique genotypes (those that did not belong to a redundant bin) were excluded from bin-map construction. As a result, 855 bins were identified with mean size ~440 kb and used for construction of bin map using the BIN function in IciMapping QTL version 4.2 (Meng et al., 2015). Allelic genotyping datasets containing two bin genotypes (Q9311B type, and WSSM type) at each of 855 bins for the 1061 RILs, and the genotype for each BC_1_F_1_ hybrid were deduced from the gene-based allelic genotypes of its parental RIL and Q9311B. Specifically, for each of the 13,847 genes, if the parents (RIL and Q9311B) have the same allelic genotype, their BC_1_F_1_ hybrid should be the homozygous genotype of Q9311B; if the parents had different homozygous genotypes, the allelic genotype of their BC_1_F_1_ hybrid was deduced as the heterozygote; and if RIL parent had the heterozygous genotype, the BC_1_F_1_ was treated as missing. The bin genotypes were used for QTL mapping.

Considering that segregation of restoring gene in RIL population can result in a difference in fertility in BC_1_F_1_ population derived from Q9311A x RILs, we speculated a RIL had restoring gene if its test-cross (BC_1_F_1_) with Q9311A had more than 70% fertility. Based on this criterion, 793 and 797 RILs were selected as restorer lines in HF and NN, respectively, because of their corresponding BC_1_F_1_ having fertility of more than 70%. So, QTL mapping for four traits (three grain shape traits and TGW) was performed separately for 1061 RIL and the corresponding BC_1_F_1_ and H_MP_ populations evaluated in both environments, while QTL mapping of GYP was separately conducted using 793 and 797 RILs with restoring ability and their corresponding BC_1_F_1_ and H_MP_ populations in HF and NN, respectively. The H_MP_ population was produced by estimation of mid-parental heterosis for each BC_1_F_1_ as follows, the mid-parental heterosis value, H_MP_ (%) = (F_1_−MP)/MP×100, where F_1_ is the trait value of BC_1_F_1_, and MP is the mean value of the corresponding paternal RIL and Q9311B (Larièpe et al., 2012). All traits were analyzed using the BIP (biparental populations) function in IciMapping QTL. The population settings of RILs and BC_1_F_1_s were F_1_RIL and P_2_BC_1_F_1_, respectively. The inclusive composite interval mapping of additive (ICIM-ADD) QTL method was performed to identify main-effect QTL (mQTL) using the default settings in which *P* values for entering a variable (PIN) were set at 0.001 and the scanning step was set at 1.0 cM. The inclusive composite interval mapping of the digenic epistatic (ICIM-EPI) QTL method detected possible digenic epistatic QTL (eQTL) using the default settings. The corresponding scan step and PIN for eQTL mapping were set at 5 cM and 0.0001, respectively. The (logarithm of odds) LOD threshold values 3.0 and 5.0 were determined according to 1000 permutations at a 95% confidence level to detect mQTL and eQTL, respectively (Van Ooijen, 1999). The physical positions of a QTL were retrieved based on the left and right markers of the detected interval.

### Inference of mQTL gene actions

The gene effects of heterosis can be dissected through QTL mapping using segregation populations such as the RILs derived from QYSM and the BC_1_F_1_s between Q9311A and the RILs, where the additive effect (*a*) of mQTL can be detected in the RILs while the sum of additive effect and dominance effect (*a*+*d*) detected in BC_1_F_1_s. When H_MP_s are used for QTL analysis of heterosis, the inherited effect should be a dominance effect (*d*). The identified mQTL can be divided into four types in RILs and BC_1_F_1_s according to Mei et al. (2005) and Kim et al. (2017). Thus, when a QTL was observed only in the RILs or BC_1_F_1_s, it was called additive effect (A). When QTL were simultaneously detected in the RILs and BC_1_F_1_s, or in the H_MP_ of BC_1_F_1_s, QTL with |2*d*/(*a* + *d*)|<1 or |2*a*/(*a* + *d*)|>1 were designated as incomplete dominant QTL (ID). QTL with |2*d*/(*a* + *d*)|>1 or |2*a*/(*a* + *d*)|<1, or those detected only in H_MP_ datasets were designated as overdominant QTL (OD), and QTL showed negative values were defined as underdominant QTL (UD). Only ID, OD, and UD mQTL were used for subsequent heterosis analysis of the detected mQTL.

### Identification of candidate genes

QTL with heterotic effects simultaneously detected in both environments for grain shape, grain weight, and grain yield traits were used for candidate gene analysis. All genes in each QTL interval were identified for further candidate-gene analysis using haplotype analysis of 732 accessions (386 *indica*/*xian*, 219 *japonica*/*geng*, 46 of intermediate type, 67 *aus*/*boro*, and 14 *basmati*/*sadri* accessions) from the 3K Rice Genome Project (3K RGP) (**Supplementary Table 1**). Grain shape, TGW and GYP phenotypic data were collected in Sanya. All available high-quality SNPs with a minor-allele frequency of more than 0.05 and a missing rate of less than 20% located in these genes were retrieved from 18 M SNP data generated from 3K RGP in the Rice SNP-Seek Database (Wang et al., 2018). Haplotype analysis was conducted for each candidate gene in each QTL region using all non-synonymous SNPs located inside the gene CDS region. Haplotypes shared by more than 10 accessions were compared. For a single candidate gene, the haplotype with the best phenotype of the corresponding trait was assigned as a favorable haplotype.

### Statistical analysis

Differences in the mean phenotypic values among the haplotypes were evaluated by one-way ANOVA by the agricolae package in R (de Mendiburu, 2021). Phenotypic differences among the check parents and the relative hybrids were assessed by Duncan’s multiple comparison test, and among RILs and the BC_1_F_1_s by Student’s *t*-test. H_MP_ was tested with a Student’s *t*-test based on the contrast between the F_1_ hybrid mean and the mean performance of the corresponding parental lines (Larièpe et al., 2012). Phenotypic correlation analyses of the five traits were computed using the corrplot package in R. Broad-sense heritabilities (*H^2^*) were calculated using the AOV module implemented in IciMapping QTL.

## Results

### Phenotypic performances

**Table 1** showed the field performance and broad-sense heritability of parental population (Q9311B and WSSM), QYSM (F_1_), the RILs, the BC_1_F_1_s (Q9311A×RILs), and the corresponding H_MP_s in HF and NN for grain shape/yield traits. Two parents, Q9311B and WSSM, are slender grain rice varieties with similar grain weight and yield except that WSSM showed significantly lower GW and GL but larger GLWR than Q9311B in HF and NN, respectively. The H_MP_s of QYSM was 4.45% and 4.48% for GL, 2.25% and 3.44% for GW, 1.72% and 0.46% for GLWR, 0.91% and 16.73% for TGW, and 8.69% and 3.00% for GYP in HF and NN, respectively, showing significant heterosis in TGW in NN. Both RIL and BC_1_F_1_ populations displayed wide segregations in five traits, particularly TGW and GYP in both environments. Comparing to QYSM, we found lower heterosis in BC_1_F_1_ population for GL, GLWR, and GYP in the two environments and GW in HF except GW in NN and TGW in the two environments, demonstrating hybrid breakdown in the RILs in GL, GLWR, and GYP because of inbreeding depression, as RILs of QYSM showed GL (96.46 and 96.55)%, GLWR (99.35 and 98.73)%, and GYP (88.26 and 96.25)% in HF and NN, respectively. The broad-sense heritabilities (*H^2^*) of RILs (BC_1_F_1_s) were 0.99 and 0.99 for each of GL, GW, and GLWR, 0.55 and 0.68 for TGW, and 0.24 and 0.48 for GYP in HF and NN, respectively (**Table 1**). Thus, the environment strongly affected the grain traits, particularly TGW and GYP.

**Table 1:** Phenotypic performance and broad-sense heritability of grain shape and grain weight as well as grain yield traits in the parent lines, QYSM (Q9311A × WSSM), RILs, BC_1_F_1_s (Q9311A × RILs), and their H_MP_s in Hefei (HF) and Nanning (NN).

Trait^a^	Env.	Q9311B^b^	WSSM	QYSM		RILs			BC_1_F_1_s			
				**F_1_**	H_MP_ (%)^c^	**Mean ± SD**	**CV (%)**	*H^2^* ^d^	**Mean ± SD**	**CV (%)**	***H^2^***	**H_MP_ (%)**
GL (mm)	HF	6.31^b^	6.13^c^	6.50^a^	4.45	6.27 ± 0.32	5.12	0.99	6.44 ± 0.24	3.69	0.99	2.42 ± 3.43
	NN	6.45^b^	6.29^c^	6.66^a^	4.48	6.43 ± 0.33	5.12		6.56 ± 0.24	3.69		1.98 ± 3.45
GW (mm)	HF	2.25^a^	1.91^c^	2.13^b^	2.25	2.07 ± 0.10	4.91	0.99	2.21 ± 0.06	2.91	0.99	2.20 ± 3.13
	NN	2.10^a^	1.88^b^	2.12^a^	3.44	2.09 ± 0.10	4.56		2.21 ± 0.06	2.50		5.55 ± 2.96
GLWR	HF	2.81^c^	3.22^a^	3.07^b^	1.72	3.05 ± 0.21	7.05	0.99	2.93 ± 0.15	5.13	0.99	0.16 ± 4.96
	NN	3.10^b^	3.35^a^	3.14^b^	0.46	3.10 ± 0.22	7.07		2.98 ± 0.15	4.97		-3.66 ± 4.66
TGW (g)	HF	30.88^a^	27.56^a^	29.49^a^	0.91	25.37 ± 2.58	10.17	0.55	27.71 ± 2.03	7.32	0.68	2.90 ± 7.12
	NN	27.60^ab^	23.97^b^	30.10^a^	16.73*	28.13 ± 2.94	10.45		32.17 ± 4.48	13.93		17.55 ± 16.81
GYP (g)	HF	26.03^a^	26.03^a^	28.29^a^	8.69	24.97 ± 3.90	15.61	0.24	24.70 ± 6.06	24.54	0.48	-3.02 ± 6.30
	NN	41.32^a^	44.05^a^	43.97^a^	3.00	42.32 ± 8.01	18.92		43.18 ± 10.77	24.94		-1.44 ± 11.92

a GL, grain length; GW, grain width; GLWR, grain length-width ratio; TGW, 1000-grain weight; GYP, grain yield per plant.

b Different letters represent significant differences at P < 0.05 based on Duncan’s multiple range test for the same traits among the two parents and QYSM.

c ^*^represents significant difference at P < 0.05 based on t-test for the same traits between F_1_ and the mean of the two parents.

d H² represents broad-sense heritability of traits.

The significant association among different traits (grain shape, weight, and grain yield) of RILs and BC_1_F_1_s under the two locations was identified by correlation analysis (**Table 2**). Under both environments, we found significant (positive and negative) correlations between GLWR and GL, and GLWR and GW in RILs and BC_1_F_1_s. Whereas, GL showed significant negative correlation with GW in BC_1_F_1_s but not in RILs. Similarly, TGW showed significant positive correlations with GL and GW in RILs but not in BC_1_F_1_s under NN, demonstrating that few correlations were related to genetic background. However, other correlations were linked to the environment. For example, TGW showed positive correlations with GL and GW in BC_1_F_1_s only in HF but not in NN, and it was true for a negative correlation between GYP and GL only in HF in BC_1_F_1_s. Whereas, all traits (GL, GW, and GLWR) presented significantly positive correlations in both RILs and BC_1_F_1_s between the two environments, but TGW only in RILs and GYP only in BC_1_F_1_s, indicating that GL, GW, and GLWR exhibited that their performance did not affect by the background and the environment.

**Table 2:** Correlations between grain shape and grain weight as well as grain yield traits estimated in RIL population and BC_1_F_1_ (Q9311A × RILs) population in Hefei (upper triangular) and Nanning (lower triangular).

Trait	GL	GW	GLWR	TGW	GYP
RIL population					
GL	0.80**	-0.01	0.72**	0.65**	0.08
GW	-0.12	0.78**	-0.69**	0.47**	0.05
GLWR	0.78**	-0.71**	0.82**	0.14	0.02
TGW	0.29**	0.40**	-0.04	0.43**	0.07
GYP	0.12	0.07	0.03	0.07	0.01
BC_1_F_1_ population					
GL	0.68**	-0.25*	0.86**	0.31*	-0.20*
GW	-0.26*	0.61**	-0.71**	0.22*	-0.09
GLWR	0.88**	-0.69**	0.69**	0.14	-0.09
TGW	0.18	0.14	0.12	0.07	-0.18
GYP	-0.16	0.01	-0.16	-0.02	0.38**

The values on principal diagonal indicated correlations between Hefei and Nanning. The values were correlation coefficients (r). The * and ** indicated significant correlation at P < 0.05 and P < 0.01, respectively. GL, grain length; GW, grain width; GLWR, grain length-width ratio; TGW, 1000-grain weight; GYP, grain yield per plant.

### Main-effect QTL (mQTL) for grain shape, weight and yield traits

**Figure 1:**
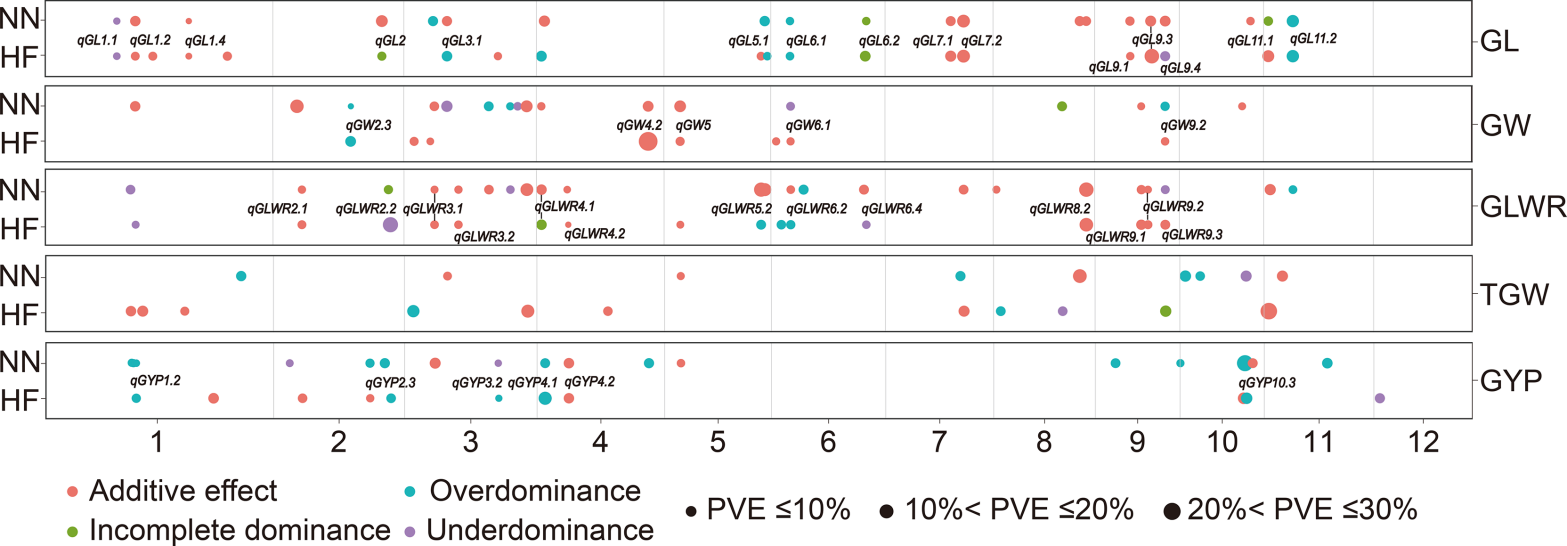
Genomic distributions of 114 mQTL for grain shape and grain weight as well as grain yield traits identified in the RILs, BC_1_F_1_s, and H_MP_s in Hefei (HF) and Nanning (NN). QTL represented in the figure were those stably identified in both environments. GL, grain length; GW, grain width; GLWR, grain length-width ratio; TGW, 1000-grain weight; GYP, grain yield per plant.

We constructed 855-bin linkage map for RIL population of about 2,268 cM, with chromosome length ranging from 106 cM (chromosome 7) to 334 cM (chromosome 2) (**Supplementary Table 2**). About 114 mQTL were identified for different grain traits (shape, weight, and yield) of RILs, BC_1_F_1_s, and corresponding H_MP_s in NN and HF by employing genotypic datasets at 855 bins for the 1,061 RILs, BC_1_F_1_s and H_MP_s. Among 114, we found 39 in both locations (**Figure 1** **;** **Supplementary Table 3**).

**Figure 2:**
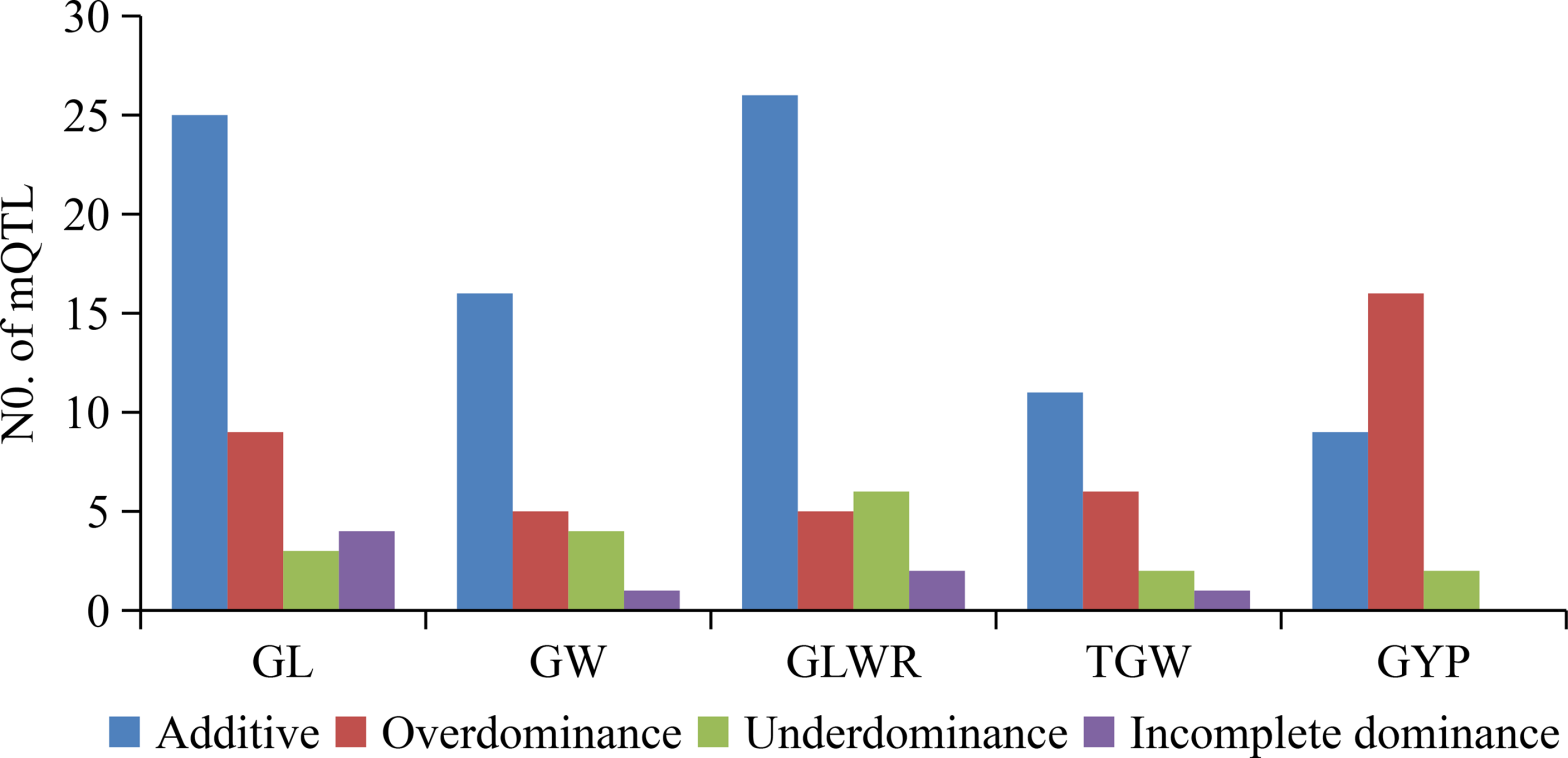
Numbers of mQTL classified by gene action for grain shape and grain weight as well as grain yield traits detected in RILs, BC_1_F_1_s, and H_MP_s. GL, grain length; GW, grain width; GLWR, grain length-width ratio; TGW, 1000-grain weight; GYP, grain yield per plant.

For four grain type and weight traits, total (21, 20) mQTL were detected in HF and NN for GL, explaining (34.0, 25.0)%, (35.6, 33.3)%, and (18.4, 16.3)% of total phenotypic variances in the RILs, BC_1_F_1_s, and H_MP_s (**Supplementary Table 3**). These 41 mQTL included additive (25), over-dominance type (OD-t, 9), under-dominance type (UD-t, 3), and incomplete-dominance type (ID-t, 4) (**Figure 2**). Among them, 15 mQTL were identified in both environments, such as three (*qGL1.4*, *qGL7.1*, and *qGL7.2*) in RILs, two (*qGL9.1* and *qGL9.3*) in BC_1_F_1_s, and two (*qGL1.1*, *qGL6.1*) in H_MP_s with the same gene actions. While, eight QTL (*qGL1.2*, *qGL2*, *qGL3.1*, *qGL5.1*, *qGL6.2*, *qGL9.4, qGL11.1*, and *qGL11.2*) were simultaneously detected in two or three populations in different environments with same or different gene actions. For GW, 9 (HF) and 17 (NN) mQTL were identified, explaining (0 HF, 10.1 NN)%, (31.8 HF, 31.2 NN)%, and (5.5 HF, 13.9 NN)% of total phenotypic variance in the RILs, BC_1_F_1_s, and H_MP_s (**Supplementary Table 3**). These 26 mQTL contained 16 additives, 1 ID-t, 4 UD-t, and 5 OD-t (**Figure 2**). Among them, 5 mQTL were concurrently seen in both HF and NN, including *qGW2.3* in H_MP_s, *qGW4.2*, and *qGW5* in BC_1_F_1_s, while *qGW6.1* and *qGW9.2* were found in BC_1_F_1_s (HF), and BC_1_F_1_s and H_MP_s in NN with different gene actions. A total of 16 (HF) and 23 (NN) mQTL for GLWR were identified, accounting for (13.9 HF, 25.5 NN)%, (38.0 HF, 42.8 NN)%, and (12.3 HF, 8.2 NN)% of the total phenotypic variance in the RILs, BC_1_F_1_s, and corresponding H_MP_s, respectively (**Supplementary Table 3**). These 39 mQTL comprised 26 additive, 2 ID-t, 6 UD-t, and 5 OD-t (**Figure 2**). Thirteen mQTL were commonly present in both environments, from which 7 mQTL, including (*qGLWR2.1*, *qGLWR3.1*, *qGLWR3.2*, *qGLWR4.2*, *qGLWR9.1*, and *qGLWR9.2*) were identified in the BC_1_F_1_s, and one mQTL (*qGLWR8.2*) in the RILs of HF and NN with same gene action. However, the other 5 mQTL (*qGLWR4.1*, *qGLWR5.2*, *qGLWR6.2*, *qGLWR6.4*, and *qGLWR9.3*) were observed in two or three populations in both locations (HF and NN) with different gene actions. About TGW, 11 (HF) and 9 (NN) mQTL were identified, explaining (37.3 HF, 4.1 NN)%, (20.5 HF, 25.6 NN)%, and (7.9 HF, 16.1 NN)% of total phenotypic variance in the RILs, BC_1_F_1_s, and H_MP_s (**Supplementary Table 3**). These 20 mQTL contained 11 additive, 1 ID-t, 2 UD-t, and 6 OD-t (**Figure 2**). No any common QTL for TGW was detected in the two environments.

For GYP, 13 (HF) and 14 (NN) mQTL were detected, explaining (0 HF, 8.7 NN)%, (45.8 HF, 27.7 NN)%, and (38.8 HF, 37.9 NN)% of total phenotypic variance in the RILs, BC_1_F_1_s, and the corresponding H_MP_s (**Supplementary Table 3**). These 27 mQTL contained 9 additive, 2 UD-t, and 16 OD-t (**Figure 2**). Among these, 6 mQTL were simultaneously detected in both HF and NN locations, including *qGYP4.1* and *qGYP4.2* detected in H_MP_s and BC_1_F_1_s, respectively, *qGYP3.2* and *qGYP10.3* in both locations of BC_1_F_1_s and H_MP_s. However, *qGYP1.2* was seen in BC_1_F_1_s and H_MP_s in HF, and H_MP_s in NN, while *qGYP2.3* seen in BC_1_F_1_s in HF, and RILs and BC_1_F_1_s in NN with differing gene actions.

Furthermore, 27 mQTL (additive effects) were found in the RILs, 97 (additive and dominant effects) were found in BC_1_F_1_s, and 54 (dominant effects) were observed in H_MP_s (**Supplementary Table 3**). However, there was only one mQTL (*qGL9.4* in HF) found between the RILs and H_MP_s, and among 11 mQTL, 4 (*qGL2*, *qGL4.1*, *qGLWR4.1*, and *TGW9*) in HF, 6 (*qGL6.2*, *qGL11.1*, *qGW8*, *qGLWR9.3*, *qGYP1.1*, and *qGYP2.3*) in NN, and one mQTL (*qGLWR2.2*) present both in HF and NN between RILs and BC_1_F_1_s, indicating that most of additive loci in the RILs did not exhibit heterotic effects as they did not simultaneously find in H_MP_s. The fixed favorable allele (FA) from WSSM or Q9311 from these 11 mQTL affect BC_1_F_1_s performance but did not show a significant heterotic effect. Twenty-five mQTL were commonly observed between BC_1_F_1_s and H_MP_s, which recommended that these loci affect the performance of BC_1_F_1_s and also contributed to heterosis.

### Epistatic QTL for grain shape, weight and yield traits

In total, 73, 164, and 122 eQTL pairs were observed in RILs, BC_1_F_1_s, and H_MP_s (**Supplementary Tables 4**, **5**). Among these, 3, 36, and 11 pairs involved one mQTL and the others a random locus in the RILs, BC_1_F_1_s, and H_MP_s, respectively. In addition, 2 pairs involved two mQTL were found in BC_1_F_1_s in NN. The total phenotypic variation explained (PVE) of eQTL was 21.6%, 65.7%, and 27.3% (GL), 23.3%, 55.2%, and 18.3% (GW), 24.4%, 61.7%, and 32.8% (GLWR), 18.4%, 30.1%, and 25.1% (TGW), and 14.9%, 18.2%, and 6.1% (GYP) in HF in the RILs, BC_1_F_1_s, and H_MP_s. In NN, 28.9%, 53.8%, and 29.6% (GL), 24.7%, 49.6%, and 13.1% (GW), 25.3%, 56.9%, and 30.2% (GLWR), 10.9%, 24.4%, and 24.4% (TGW), and 15.3%, 1.6%, and 0% (GYP) were found in RILs, BC_1_F_1_s, and H_MP_s, implying that epistasis is a key factor in heterosis. About 23.64% of common epistasis was detected for TGW between BC_1_F_1_s and H_MP_s in the two locations, which is significantly higher than 4.35% (GL), 0% (GW), 3.80% (GLWR), and 0% (GYP), demonstrating that epistasis displayed higher contribution in grain weight with heterotic effect as compared to grain type and yield traits in BC_1_F_1_s.

### QTL regions simultaneously affecting grain shape, weight and yield traits

**Figure 3:**
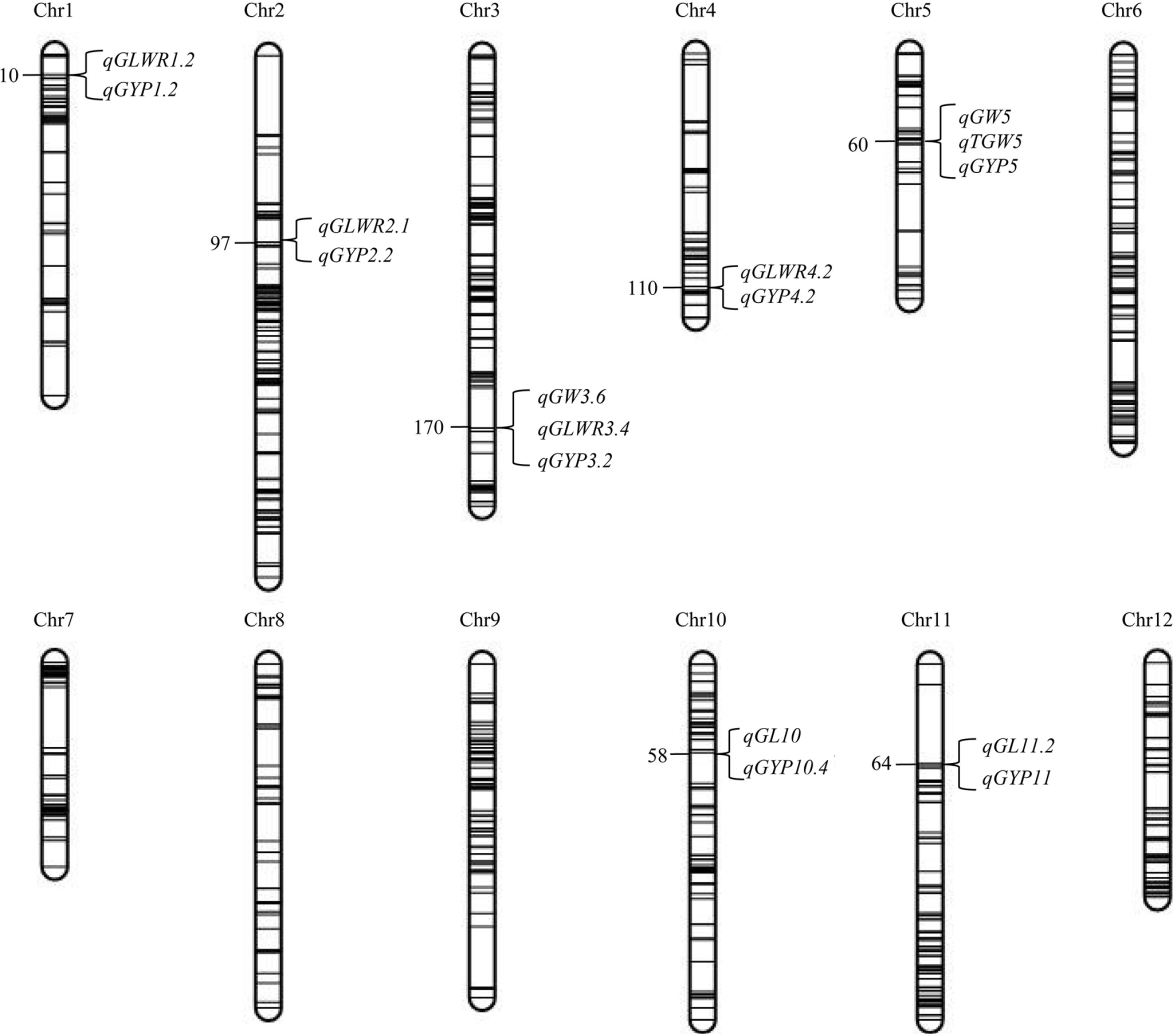
mQTL affecting grain shape and grain weight as well as grain yield traits detected in BC_1_F_1_s and the corresponding H_MP_s in Hefei (HF) and Nanning (NN). Number on the left side of the chromosome indicates the genetic position of the QTL in cM. The linkage map spans 2,268 cM with a mean of 2.65 cM between neighboring bins.

We found seven QTL regions on chromosomes (chr) 1, 2, 3, 4, 5, 10, 11 that simultaneously affected the grain shape, grain weight, and grain yield traits under the same environment (**Figure 3** **;** **Supplementary Table 6**). Five regions were found with opposite directions of FA effects of grain shape, weight, and grain yield traits, including the region of 6463296−6583687 bp on chr1, containing *qGLWR1.2* and *qGYP1.2* found in BC_1_F_1_s, and H_MP_s in HF; the region of 28248757−30003190 bp on chr3, harboring *qGYP3.2* identified in BC_1_F_1_s and H_MP_s, while *qGW3.6* and *qGLWR3.4* detected in H_MP_s in NN; the region of 5111957−6692156 bp on chr4 harboring *qGLWR4.2* and *qGYP4.2* detected in BC_1_F_1_s in NN; the region of 4578959−5986015 bp on chr5 harboring *qGW5*, *qTGW5* and *qGYP5* detected in BC_1_F_1_s in NN; and the region of 20001168 −20792875 bp on chr10 harboring *qGL10* and *qGYP10.4* detected in BC_1_F_1_s in NN. However, we found two regions with the consistent direction of FA effects for grain traits (type and yield), including 8135122−8389827 bp on chr2, containing *qGLWR2.1* and *qGYP2.2* found in BC_1_F_1_s in HF, and the region of 8243782−9022284 bp on chr11 harboring *qGL11.2* detected in H_MP_s, while *qGYP11* detected in BC_1_F_1_s and H_MP_s in NN. Based on this, we assume that a partial genetic overlap is existed between different grain traits (shape, weight, and yield) either in same or opposite directions of FA for each mQTL.

### Candidate genes affecting grain shape, weight and grain yield

Among 39 mQTL affecting rice grain (shape, yield) traits in both environments (**Supplementary Table 7**), 12 mQTL had heterotic effects without previous cloned genes. Based on haplotype analysis, there was no significant difference in haplotypes among *qGLWR2.2*, *qGYP4.1*, and *qGL11.1*. In the remaining 9 mQTL, 19 candidate genes with haplotype differences were detected. While, two different populations *(xian, geng)* were used for phenotypic characterizations of the haplotypes (**Supplementary Table 8**).

**Figure 4:**
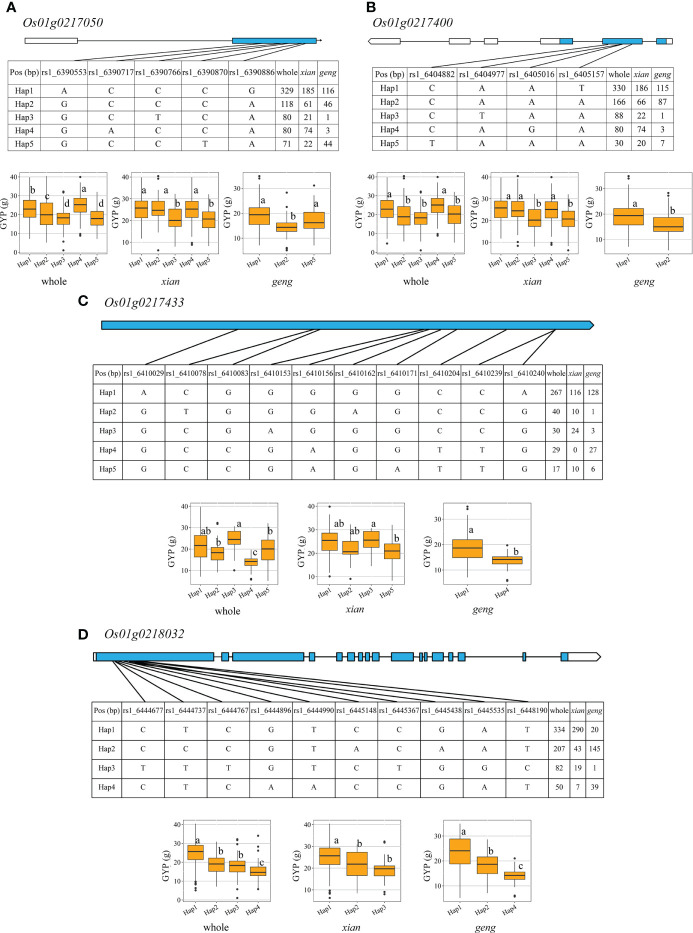
Exon-intron structure and haplotype analysis of four candidate genes in whole, *xian* and *geng* populations for *qGYP1.2.* **(A)** *Os01g0217050*, **(B)** *Os01g0217400*, **(C)** *Os01g0217433*, **(D)** *Os01g0218032*. Different letters indicate significant differences in trait values among haplotypes by Duncan’s test at *p* < 0.05.

We found four candidate genes (*Os01g0218032, Os01g0217050, Os01g0217433*, and, Os01g0217400) for *qGYP1.2* (**Figure 4** **;** **Supplementary Table 8**). Five and three haplotypes were found in both (*xian*, *geng)* populations for *Os01g0217050.* Besides, Hap 1 showed profound increase in GYP but decrease GL, GW, GLWR and TGW only in *geng* population. Similarly, five and two haplotypes were found in both populations for *Os01g0217400.* While Hap 1 showed significant increase in GYP but decrease GL, GLWR and TGW only in *geng* population. In *xian* population, both genes *Os01g0217050* and *Os01g0217400* had prominent effects on GYP, GL, GW and GLWR but did not show any effect on TGW. Four and two haplotypes were found in both populations for *Os01g0217433*, and Hap 3 markedly increased GYP, GL, and GLWR only in *xian* population. In contrast, Hap 1 markedly increased GYP but decreased GL, GW, and TGW only in *geng* population. Three haplotypes were found in both populations for *Os01g0218032*, and Hap 1 enhanced GYP but decreased GL, GW, and TGW only in *geng* population. Further, *Os01g0218032* did not show any noticeable effect on grain shape/weight traits in *xian* population.

Four candidate genes (*Os02g0670000*, *Os02g0670400*, *Os02g0671100*, and *Os02g0671800*) were observed for *qGYP2.3* (**Supplementary Table 8**). Two haplotypes for *Os02g0670000, Os02g0670400* and *Os02g0671100* were found in both populations. The favorable Hap 1 in two populations markedly enhanced GYP but did not exhibit any effect on TGW in *xian* population, while showed substantial effect on GW in *geng* population. In *xian* population, five haplotypes were observed for *Os02g0671800* and the Hap 4 had considerably enhanced GYP, GL, and GLWR. No haplotype for *Os02g0671800* was detected in the *geng* population.

For *qGYP3.2*, four candidate genes (*Os03g0715600*, *Os03g0716400*, *Os03g0717600*, and *Os03g0719500*) were identified only in *xian* population (**Supplementary Table 8**), suggesting this gene is specific to *xian* subspecies. Four (three) haplotypes were observed for *Os03g0715600* (*Os03g0717600*), and Hap 2 considerably enhanced GYP, GL, and GLWR but decreased GW and TGW in *xian* population. Both *Os03g0716400* and *Os03g0719500* contain two and four haplotypes, and Hap 2 considerably enhanced GYP, GL, and GLWR but did not show any effect on TGW in *xian* population.

**Figure 5:**
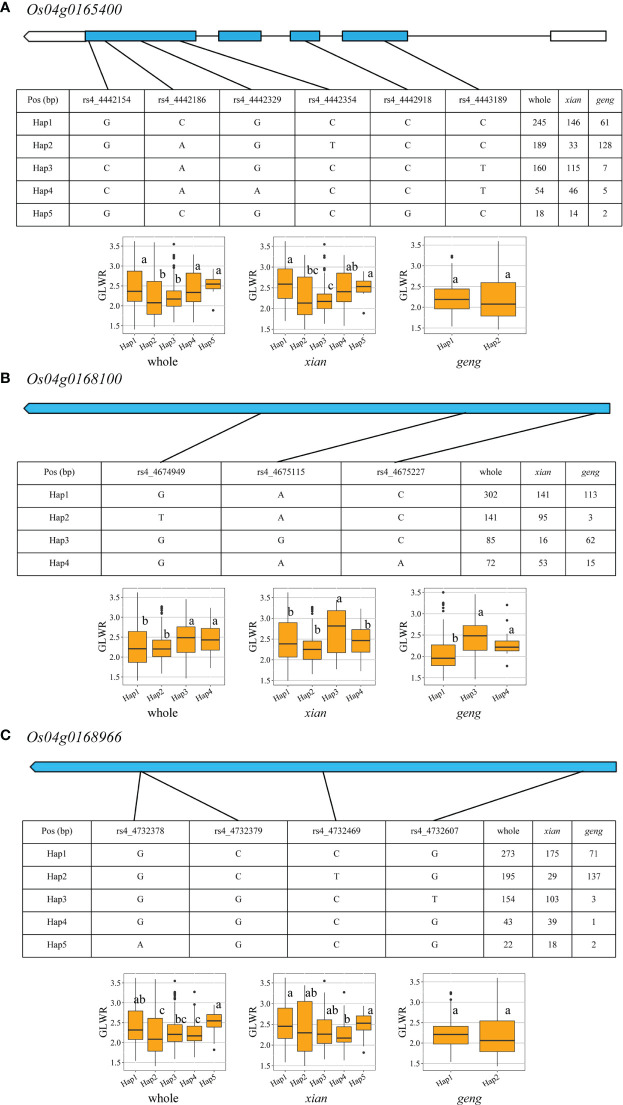
Exon-intron structure and haplotype analysis of three candidate genes in whole, *xian* and *geng* populations for *qGLWR4.1.* **(A)** *Os04g0165400*, **(B)** *Os04g0168100*, **(C)** *Os04g0168966*. Different letters indicate significant differences in trait values among haplotypes by Duncan’s test at *p* < 0.05.

For *qGLWR4.1*, three candidate genes (*Os04g0165400*, *Os04g0168100*, and *Os04g0168966*) were observed (**Figure 5** **;** **Supplementary Table 8**). Five and two haplotypes were found in both populations for *Os04g0165400*, the favorable Hap 1 considerably enhanced GLWR, GL, TGW, and GYP but decreased GW in the *xian* population. In *geng* population, *Os04g0165400* showed prominent effect on GL, TGW, and GYP. Four and three haplotypes were found in both populations for *Os04g0168100*, and Hap 3 had noticeable effect on GL, GW, GLWR, and GYP in the two populations. Five and two haplotypes were found in both populations for *Os04g0168966*, and Hap 1 had significant effect on GW, GYP, GLWR, and TGW only in *xian* population. Whereas, *Os04g0168966* presented noticeable effect on GYP, TGW, and GL only in *geng* population.

Two candidate genes (*Os05g0567500* and *Os05g0569300*) were detected for *qGL5.1* and *qGLWR5.2* (**Supplementary Table 8**). Whereas, we found only two haplotypes in *geng* population, suggesting this gene is specific to *geng* subspecies. Hap 1 had significant effect on GL, GW, and GLWR for *Os05g0567500* and *Os05g0569300* in the *geng* population.

For *qGL6.1*, *qGW6.1*, and *qGLWR6.2*, two candidate genes (*Os06g0214900* and *Os06g0215200*) were identified (**Supplementary Table 8**). Three haplotypes for *Os06g0214900* and two haplotypes for *Os06g0215200* were observed in the two populations. A considerable effect was observed in GL, GW, GLWR, and GYP for Hap 3 of *Os06g0214900* in both populations. For *Os06g0215200*, Hap 2 presented noticeable effect on GL, GLWR, and GYP in *xian* population, and GL, GW, GYP, and GLWR in *geng* population.

These findings indicated that many candidate genes have pleiotropic effects with different favorable haplotypes between these two populations, verifying that these genes are linked with variations in grain traits (shape, yield) between the two subspecies.

## Discussion

The simultaneous improvement of cereal crops grain quality and yield is a main challenge in modern agriculture. Grain weight and grain type traits that contribute to grain yield greatly influence the commercial value of rice (Zheng et al., 2007; Qiu et al., 2015). Nowadays, breeding objectives of hybrid rice have been transferring from high yield to synergy of high yield and premium quality. So, it is vital to dissect genetic mechanisms of heterosis and their genetic relationships for effectively developing rice hybrid with good grain quality and high yield. QYSM is a best choice for genetic dissection of heterosis for rice grain traits.

### Genetic dissection of heterosis for grain type, weight and grain yield

Despite the extensive reports on heterosis (Chu et al., 2012; Kim et al., 2017; Yang et al., 2021), reports about heterosis of grain traits are very rare. In this work, single-locus overdominance plays a critical role in rice yield heterosis (**Figure 2**), in accordance with the results reported by Melchinger et al. (2007) and Li et al. (2015). Contrarily, single-locus overdominance and epistasis affected rice grain shape heterosis (**Figure 2**; **Supplementary Tables 4**, **5**). Five OD-t mQTL (*qGL3.1*, *qGL11.2*, *qGW2.3*, *qGLWR6.3*, and *qGLWR11.2*), and two ID-t mQTL (*qGL2* and *qGL6.2*) were detected as heterotic loci for grain shape and mapped together with six known genes related to grain shape, i.e., *SRL2* (Liu et al., 2016), *SRS5* (Segami et al., 2012), *SGL1* (Wang et al., 2016), *TGW2* (Ruan et al., 2020), *GL6* (Wang et al., 2019a), and *GW6a* (Song et al., 2015) (**Supplementary Table 3**), suggesting above cloned genes may have heterosis for grain type traits. The moderate rolled leaf is ideal for rice phenotype as it is helpful to attain exemplary plant architecture, consequently improving photosynthesis efficiency and grain yield (Wu, 2009). *SRL2* gene plays a crucial role in regulating leaf development, and its deficiency leads to inwardly rolled leaves (Liu et al., 2016). Furthermore, grain type and grain weight are mainly correlated to rice yield potential. *SRS5* identified with an overdominance effect on heterosis of GL, GLWR, and GYP promotes the rice grain size (Si et al., 2016; Segami et al., 2017), and positively regulates the GL through cell elongation (Segami et al., 2012). Similarly, *SGL1* also increases the GL of rice (Wang et al., 2016). *GW6a* and *GL6* regulate the grain weight, while *TGW2* negatively regulates the grain weight (Ruan et al., 2020).

Similarly, four UD-t mQTL (*qGL1.1*, *qGLWR1.1*, *qGL9.4* and *qGLWR9.3*) and three OD-t QTL (*qGW9.2*, *qTGW8.1*, and *qGYP10.3*) were detected for heterosis of grain shape, weight, and grain yield and mapped together with the known genes related to grain yield like *D2* (Dong et al., 2016), negative regulators of grain yield gene *Gnla* (Ashikari et al., 2005; Li et al., 2016), grain width, and weight *GW5L* (Tian et al., 2019), and two other cloned genes related to grain type, plant height, and grain numbers, such as *OsBC1* (Jang et al., 2017), and *Ghd8* (Wang et al., 2019b). Tiller angle is a vital part of plant architecture that significantly affects the grain yield. A tiller angle-related gene *D2* positively improves rice plant architecture by controlling tiller angle and enhancing grain yield (Dong et al., 2016). However, *GW5L* and *Gnla* negatively regulate the rice grain width, grain weight, and production, respectively (Li et al., 2016; Tian et al., 2019). In contrast, two genes, *Ghd8* and *OsBC1*, positively regulate the grain number, grain size, and plant height (Jang et al., 2017; Wang et al., 2019b). Thus, these biological function genes influencing different grain traits may assess the genetic effect on heterosis of grain shape and yield.

**Supplementary Table 5** indicated higher epistasis for grain weight than grain shape quality. Epistasis was compared between BC_1_F_1_s and H_MP_s in two environments, and more epistasis was found between BC_1_F_1_s and H_MP_s for grain weight than three grain shape and grain yield traits, indicating that epistasis had more contribution to heterosis of grain weight than grain shape and grain yield traits. By considering the number of mQTL identified for different grain traits, it is concluded that heterosis for TGW was mainly controlled by epistasis while GL, GW, GLWR, and GYP was mainly controlled by UD or OD, indicating that the genetic mechanisms is different for both grain shape and grain yield, and grain weight.

### Effect of environment on grain shape, weight and grain yield heterosis

Crop yield heterosis is mainly affected by environment (Li et al., 2015; Lin et al., 2020a; Lin et al., 2020b). Previous study also revealed that the heterotic loci for yield were mainly affected due to genetic background and environmental conditions (Li et al., 2018). However, the behavior of environmental factors affecting rice’s heterosis is still uncertain. The current research showed a lower proportion (55%) of environment-specific mQTL for grain shape heterosis compared to yield heterosis (78%), while a high proportion (100%) of mQTL for grain weight heterosis was observed. Thus, the impact of the environment on heterotic loci of grain shape is considerably different from grain weight and yield. Such as *qGL2*, *qGL3.1*, *qGL9.4*, *qGLWR4.1*, *qGLWR5.2*, *qGLWR6.2*, and *qGLWR6.4* were detected as the heterotic loci in HF but with additive effect in NN, while opposite results were observed in *qGL5.1*, *qGL11.1*, *qGW6.1*, *qGW9.2*, *qGLWR9.3*, and *qGYP2.3* (**Supplementary Table 3**). Additionally, most eQTL were identified for heterosis of environment-specific grain shape traits (**Supplementary Table 5**). These results suggested that the environmental conditions affected heterotic loci of grain shape and weight in a different way.

### Comparisons of detected grain quality mQTL with previously known genes

Rice grain quality affected by mQTL that was simultaneously found in both locations were compared with already reported genes present at the same or adjacent to the physical location. (**Figure 1**; **Supplementary Table 3**). Some identified mQTL were present near the already known genes. For example, *qGL7.2* and *qGLWR7* in the 22056455−26044575 bp region on chr 7, affecting GL in RILs under both environments, and GLWR in RILs in NN, were mapped together with *OsGASR9* for grain size and yield in rice (Li et al., 2019), and *GL7* for grain size (Wang et al., 2015). Similarly, *qGW3.8* and *qGLWR3.5* in the region of 34560598−34700840 bp on chr 3, affecting GW and GLWR in BC_1_F_1_s in NN, were mapped in the same region of *qTGW3* that negatively modulates rice grain size and weight (Ying et al., 2018). Furthermore, *qGW5*, *qGLWR5.1*, and *qTGW5* located in 4578959−5986015 bp on chr 5, affecting GW in BC_1_F_1_s under both locations, were mapped in the same region with the known gene *GW5* that controls the width and weight of rice grain (Weng et al., 2008; Liu et al., 2017). *qTGW4* located in 4578959−5986015 bp on chr 4, affecting TGW in BC_1_F_1_s in HF, was mapped with *Gnp4*, *YDA1*, and *GIFI* genes that act as positive regulators to enhance rice grain size and weight (He et al., 2017; Zhang et al., 2018). Similarly, *qGL6.2* in the region of 26554537−27705641 bp on chr 6, affecting the GL in BC_1_F_1_s and H_MP_s in HF and RILs and BC_1_F_1_s in NN was mapped in the same region with *GL6* and *GW6a*. *GL6* positively regulates the grain length and weight through stimulating the cell propagation in grains (Wang et al., 2019a), and *GW6a* also controls the rice grain traits (Song et al., 2015; Shi et al., 2020; Zhang et al., 2020). *GL6* and *GW6a* were mapped with *qGL6.2*, *qGLWR6.3*, and *qGLWR6.4* with ID, OD, and UD heterotic effects. These two genes may have heterotic effects on grain traits. However, allelism between mQTL for grain shape, weight and yield described above and previously reported genes need to be confirmed further through gene cloning and fine-mapping.

Candidate genes for nine stable mQTL (**Supplementary Table 8**) were further inferred through bioinformatics and gene expression database. For example, *qGYP1.2* contains four candidate genes, *Os01g0218032*, *repressor of silencing 1a* (*ROS1a*) gene essential for developing gametophytes (Ono et al., 2012). It was reported that any interference in *ROs1a* gene induced pollen and embryo sac defects in rice, consequently reduced the rice yield (Xu et al., 2020). Of the four candidate genes for *qGYP2.3*, only *Os02g0671100* is mainly expressed in seeds and encodes *OsFBDUF13* -F-box and DUF domain containing protein (http://rice.uga.edu/cgi-bin/ORF_infopage.cgi?orf=LOC_Os02g44990.1) that positively regulates the spikelet numbers (Kaur et al., 2018). Furthermore, F-box protein also reduced abiotic stress tolerance and promoted root growth in rice (Yan et al., 2011). For *qGYP3.2*, *Os03g0715600* encodes EF-hand family protein (http://rice.uga.edu/cgi-bin/ORF_infopage.cgi?orf=LOC_Os03g50760.1), reducing the yield loss by protecting the rice plant against biotic stress (Ye et al., 2017). *Os03g0716400* (http://rice.uga.edu/cgi-bin/ORF_infopage.cgi?orf=LOC_Os03g50790.1) and *Os03g0719500* (http://rice.uga.edu/cgi-bin/ORF_infopage.cgi?orf=LOC_Os03g51020.1) both expressed in seeds and encoded uncharacterized conserved proteins, so these both are likely candidate genes. Three candidate genes for *qGLWR4.1*, only *Os04g0168100* encodes Zinc finger, C2H2-type domain containing protein (http://rice.uga.edu/cgi-bin/ORF_infopage.cgi?orf=LOC_Os04g08600.1) that is master regulators of abiotic stress response in plants (Han et al., 2020), and particularly regulates salt and drought tolerance in rice (Huang et al., 2009). For *qGL5.1* and *qGLWR5.2*, *Os05g0569300* expressed in seeds and encodes G-box binding factor 1 (GBF1) (http://rice.uga.edu/cgi-bin/ORF_infopage.cgi?orf=LOC_Os05g49420.1), which play essential role in fast-growing pollen in higher plants (Richter et al., 2012). Two candidate genes for *qGL6.1*, *qGW6.1*, and *qGLWR6.2*, only *Os06g0215200* (http://rice.uga.edu/cgibin/ORF_infopage.cgi?orf=LOC_Os06g11170.1) is mainly expressed in seeds, and encodes formin binding protein. Formin rice morphology determinant (RMD) plays a significant role to determine morphology of rice. RMD mutants exhibit bending growth pattern in seedlings, and have aberrant panicle and seed shape. This study suggested that the rice formin protein plays a key role in assessing the morphology of plants through regulation of actin dynamics and accurate spatial organization of microtubules and microfilaments (Zhang et al., 2011; Chang et al., 2022).

### Breeding technique aimed at grain quality and high yield in hybrid rice

The prime purpose of rice breeding is to attain good quality grain and yield (Xu et al., 2015). Owing to the low heritability and genotype-by-environment interaction, it becomes quite challenging for traditional breeding techniques to improve rice yield and quality traits simultaneously. Using knowledge of rice heterosis, it is sure to produce high yield and good quality hybrid varieties by introgressing or pyramiding FAs to achieve the finest combination of the heterosis-related genes. In the same genomic regions, we have detected some mQTL for different traits, like a region on chr 5 (4.58−5.99 Mb) containing *qGW5*, *qTGW5*, and *qGYP5* (**Supplementary Table 6**). This region also contains a known grain-size gene *GW5* (Liu et al., 2017). The WSSM alleles at the detected QTL reduced GW and enhanced GYP, suggesting that *GW5* may improve grain size and yield of hybrid rice simultaneously. Likewise, it would be possible to increase grain yield and quality together by pyramiding FA with the same directions of gene effects for GYP and grain size at the QTL regions 81315122−8389827 bp on chr 2, and 28248757−3003190 bp on chr 3, and 20001168−20792875 bp on chr 10, to attain best combinations of mQTL linked with quality and yield.

Additionally, in this research, 11 identified mQTL (*qGL2*, *qGL4.1*, *qGL6.2*, *qGL11.1*, *qGW8*, *qGLWR2.2*, *qGLWR4.1*, *qGLWR9.3*, *qTGW9*, *qGYP1.1*, and *qGYP2.3*) were found in RILs and BC_1_F_1_s, but not in H_MP_s, mainly contributed to BC_1_F_1_’ performance but with negligible heterotic effect (**Supplementary Table 3**). The same FA in WSSM and Q9311B at above 11 mQTL influenced BC_1_F_1_’s performance. Twenty-five mQTL found in both BC_1_F_1_s and H_MP_s contributed to BC_1_F_1_ performances and heterosis (**Supplementary Table 3**). Therefore, further, improvement can be achieved in hybrid rice yield and quality by eliminating UD loci and pyramiding OD/D loci that are polymorphic between the parents. Additionally, the FA found in this study will provide a valuable reference for future breeding activities to simultaneously improve grain quality and yield.

Two subspecies offer noticeable differences in grain shape. The *geng-*type alleles (*qSW5/GW5*, *OsSPL16/GW8*, *GS6*) increased the width of rice grain (Wang et al., 2012; Liu et al., 2017; Chang et al., 2022), while *GS5* and *OsSPL13/GW7 of xian-*type alleles increase grain length (Li et al., 2011; Si et al., 2016). In the current study, we found many candidate genes had different phenotypic effects on grain traits and GYP between the two subspecies (**Supplementary Table 8**). So, in rice breeding for grain shape, weight and yield quality, we can transfer favorable *geng* alleles (haplotypes) of *Os05g0567500*, *Os05g0569300*, and *Os01g0218032* into *xian* background to improve grain shape quality of *xian* variety. In a similar way, we can transfer favorable *xian* alleles of *Os03g0715600*, *Os03g0717600*, *Os03g0716400*, and *Os03g0719500* into *geng* background to improve grain shape, weight, and grain yield quality of *geng* variety or pyramiding above non-allelic FA to simultaneously improve grain shape and weight quality as well as grain yield in rice breeding of *xian*-*geng* inter-subspecific heterosis.

## Data availability statement

The original contributions presented in the study are publicly available. This data can be found here: https://rfgb.rmbreeding.cn/file/upload/rfgb/SNP%20data%20of%20RILs_frmXu_20220913_Read.xlsx.

## Author contributions

HY, SZ, KC, CS, HW, and FjZ performed the experiments. SZ and HY drafted the manuscript. FZ and HY did data analysis. JX and CZ designed the experiments and revised the manuscript. All authors contributed to the article and approved the submitted version.

## Funding

This work was funded by the Hainan Yazhou Bay Seed Lab Project (B21HJ0216), the Key Research and Development Project of Hainan Province (ZDYF2021XDNY128), and the Agricultural Science and Technology Innovation Program and the Cooperation and Innovation Mission (CAAS-ZDXT202001) and Science and Technology Key Project of Changde of Hunan Province (2021-59).

## Conflict of interest

HW and FjZ are employed by Hunan Tao-Hua-Yuan Agricultural Technologies Co. LTD, and CZ is employed by Win-All Hi-Tech Seed Co., Ltd., Hefei, China.

The remaining authors declare that the research was conducted in the absence of any commercial or financial relationships that could be construed as a potential conflict of interest.

## Publisher’s note

All claims expressed in this article are solely those of the authors and do not necessarily represent those of their affiliated organizations, or those of the publisher, the editors and the reviewers. Any product that may be evaluated in this article, or claim that may be made by its manufacturer, is not guaranteed or endorsed by the publisher.
