# Supplementary figures and images for: Genetic dissection of grain traits and their corresponding heterosis in an elite hybrid
Source: Front Plant Sci. 2022 Oct 5;13:977349. doi: 10.3389/fpls.2022.977349 (PMC9581170; doi:10.3389/fpls.2022.977349)

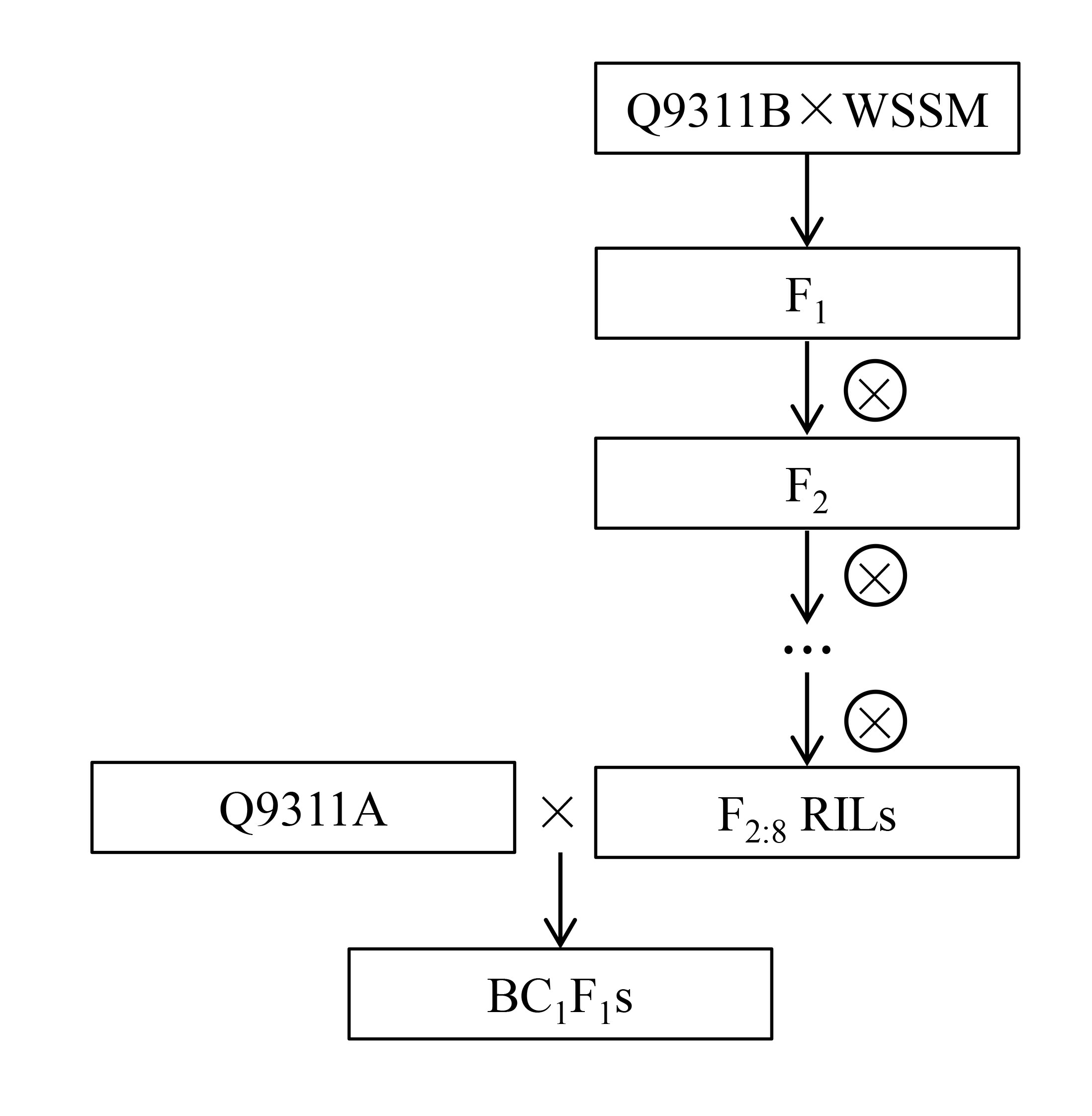

Supplement: Supplementary file 1 [file Image_1.tif]
